# Translation and cross-cultural adaptation of the Scleroderma Health Assessment Questionnaire to Brazilian Portuguese

**DOI:** 10.1590/1516-3180.2014.1323621

**Published:** 2014-04-28

**Authors:** Aline Cristina Orlandi, Fernanda Pontes Cardoso, Lucas Macedo Santos, Vaneska da Graça Cruz, Anamaria Jones, Cristiane Kyser, Jamil Natour

**Affiliations:** I MSc. Student, Rheumatology Division, Universidade Federal de São Paulo (Unifesp), São Paulo, Brazil; II MSc. Occupational therapist, Rheumatology Division, Universidade Federal de São Paulo (Unifesp), São Paulo, Brazil; III Physiotherapist, Rheumatology Division, Universidade Federal de São Paulo (Unifesp), São Paulo, Brazil; IV PhD. Student, Rheumatology Division, Universidade Federal de São Paulo (Unifesp), São Paulo, Brazil; V PhD. Physiotherapist, Rheumatology Division, Universidade Federal de São Paulo (Unifesp), São Paulo, Brazil; VI MD, PhD. Affiliated Professor, Rheumatology Division, Universidade Federal de São Paulo (Unifesp), São Paulo, Brazil; VII MD, PhD. Associate Professor, Rheumatology Division, Universidade Federal de São Paulo (Unifesp), São Paulo, Brazil

**Keywords:** Scleroderma, systemic, Quality of life, Disability evaluation, Chronic disease, Translating, Escleroderma sistêmico, Qualidade de vida, Avaliação da deficiência, Doença crônica, Tradução

## Abstract

**CONTEXT AND OBJECTIVE:**

Systemic sclerosis is an autoimmune disease characterized by abnormalities of vascularization that may cause fibrosis of the skin and other organs and lead to dysfunction. It is therefore essential to have tools capable of evaluating function in individuals with this condition. The aim of this study was to translate the Scleroderma Health Assessment Questionnaire (SHAQ) into Portuguese, adapt it to Brazilian culture and test its validity and reliability.

**DESIGN AND SETTING:**

The validation of SHAQ followed internationally accepted methodology, and was performed in university outpatient clinics.

**METHODS:**

SHAQ was translated into Portuguese and back-translated. In the cultural adaptation phase, it was applied to 20 outpatients. Items not understood by 20% of the patients were modified and applied to another 20 outpatients. Twenty patients were interviewed on two different occasions to determine the validity and reliability of the questionnaire: two interviewers on the first occasion and one interviewer 14 days later. To determine the external validity, comparisons were made with Health Assessment Questionnaire (HAQ), Disabilities of the Arm, Shoulder and Hand (DASH) and short form-36 (SF-36).

**RESULTS:**

In the interobserver evaluation, Pearson's correlation coefficient and the intraclass correlation coefficient were both 0.967. In the intraobserver evaluation, Pearson's correlation coefficient was 0.735 and the intraclass correlation coefficient was 0.687. Regarding external validity, SHAQ scores were statistically correlated with all measurements, except the general health domain of SF-36 and the work-related score (Q2) of DASH.

**CONCLUSION:**

The Brazilian version of SHAQ proved to be valid and reliable for assessing function in patients with diffuse systemic sclerosis.

## Abstract (pt)

**CONTEXTO E OBJETIVO:**

A esclerose sistêmica (ES) é uma doença autoimune caracterizada por anormalidades da vascularização, que podem gerar fibrose da pele e outros órgãos, podendo levar a disfunção. Assim, torna-se imprescindível a elaboração de instrumentos capazes de avaliar a função de indivíduos com ES. O objetivo deste estudo foi traduzir para o português, adaptar à cultura brasileira e testar a validade e confiabilidade do Scleroderma Health Assessment Questionnaire (SHAQ).

**TIPO DE ESTUDO E LOCAL:**

A validação do SHAQ seguiu uma metodologia aceita internacionalmente, e foi realizada nos ambulatórios da universidade.

**MÉTODOS:**

Foi realizada tradução e contra-tradução do instrumento. Na fase de adaptação cultural, o SHAQ foi aplicado a 20 pacientes. As questões que apresentaram mais de 20% de incompreensão foram modificadas e aplicadas em outros 20 pacientes. Para validade e confiabilidade, 20 pacientes foram entrevistados em dois momentos: no primeiro, por dois entrevistadores e após 14 dias por um deles. Foram aplicados o Health Assessment Questionnaire* (*HAQ), o Disabilities of the Arm, Shoulder and Hand (DASH) e o short form-36 (SF-36), para a validade externa.

**RESULTADOS:**

Na avaliação interobservador, o coeficiente de correlação de Pearson e o coeficiente de correlação intraclasse foram ambos de 0,967. Na avaliação intraobservador o coeficiente de correlação de Pearson foi de 0,735, e o coeficiente de correlação intraclasse foi de 0,687. Na validade externa, os escores do SHAQ foram associados estatisticamente com todos os instrumentos, exceto com o domínio estado geral de saúde do SF-36 e o escore relacionado ao trabalho (Q2) do DASH.

**CONCLUSÃO:**

A versão brasileira do SHAQ mostrou ser válida e confiável para a avaliação de função em pacientes com esclerose sistêmica difusa.

## INTRODUCTION

Systemic sclerosis is an autoimmune disease of connective tissue characterized by abnormalities in the vascular system, immune system and extracellular matrix. It can lead to fibrosis of the skin and other organs, such as the lungs, gastrointestinal tract, heart and kidneys.^1^ Systemic scleroderma is a heterogeneous disease ranging from a mild form characterized by lesser involvement of internal organs to a more severe type characterized by greater involvement of these organs and rapid progression, leading to disability and even death in some cases.^2^

Systemic sclerosis affects women more than men, in proportions of 4:1.^3^ The prevalence ranges from seven to 489 cases per million individuals. This variation has been correlated to age, region and gender.^4^ The incidence is greatest among middle-aged women and the main characteristics are Raynaud's phenomenon, thickening of the skin and dysfunction in internal organs.^5^

This disease leads to dysfunction and a reduction in life expectancy. Treatment should involve improving quality of life. It is therefore essential to have reliable assessment tools for evaluating function in individuals with systemic scleroderma. However, there are currently no specific tools validated in Brazilian Portuguese for evaluating function in such individuals.

The Health Assessment Questionnaire (HAQ)^6^ has been validated for the Brazilian Portuguese language^7^ and assesses functional capacity through 20 questions distributed among eight domains, addressing activities performed with the upper and lower limbs. The final score ranges from 0 to 3 points, such that higher scores denote worse functional capacity. This questionnaire has been widely used for evaluating function in individuals with different musculoskeletal diseases, especially rheumatoid arthritis.^8^

The Scleroderma Health Assessment Questionnaire (SHAQ) is one of the few measurement tools for evaluating function in individuals with systemic scleroderma and has been used in a number of countries.^8^ ^-^ ^10^ Like HAQ, SHAQ is made up of 20 items distributed among eight domains and has five additional domains that assess dysfunctions caused by the symptoms of systemic scleroderma. For this, five visual analogue scales (VASs) are used. The scores on these scales are converted to subscores ranging from 0 to 3 points. The overall score of the questionnaire is the sum of each of the five VAS subscores and the scores for the eight domains, divided by 13.^8^

## OBJECTIVE

The aim of the present study was to translate the Scleroderma Health Assessment Questionnaire into Brazilian Portuguese, adapt it to Brazilian culture and test its validity and reliability.

## METHODS

This study was carried out in two stages. The first stage consisted of translation of SHAQ into Brazilian Portuguese and cross-cultural adaptation to the Brazilian population. The second stage consisted of determining the reliability and external validity of the Brazilian version of SHAQ. This study was approved by our institution's Research Ethics Committee.

*Translation and cross-cultural adaptation*

The translation, validation and adaptation of the questionnaire were carried out following the method proposed by Guillemin et al.^11^ ^,^ ^12^

### Translation to the Portuguese language

SHAQ was translated independently by two teachers of English who were informed about the aim of the study. The two versions of the questionnaire in Portuguese were analyzed and compared by a team made up of two rheumatologists and three physiotherapists, who examined the questionnaire for possible translation problems and analyzed the applicability of each item. This process generated a single translated version of SHAQ (V1).

### Assessment of initial translation (back translation)

V1 was then back-translated into English by two English-language teachers who were fluent in Portuguese. In this phase, the teachers were unaware of the original questionnaire or the aim of the study. The two back-translated versions were compared with the original questionnaire by the same team, in order to determine the semantic and grammatical equivalence, ensuring the similarity between the original questionnaire and V1, which could be used as the final version of the questionnaire (**Annex**).

*Sample*

A total of 60 male and female patients between 18 and 65 years of age with systemic scleroderma were recruited from our institution's outpatient clinics. For patients to be included, they needed to fulfill the clinical criteria for the diagnosis of systemic sclerosis.^13^ Authorization was obtained from the authors of SHAQ for its use in the present study.

### Assessment of comprehension (cultural equivalence)

The final Portuguese version (V1) of SHAQ was applied to 20 patients with systemic scleroderma in order to assess the degree of comprehension of each item. All items that were considered incomprehensible or not applicable by at least 20% of the patients were revised and modified, and were then applied to 20 different patients.

### Assessment of measurement properties Reliability

Following the translation and cross-cultural adaptation of SHAQ, it was applied to a new group of 20 patients with systemic scleroderma. Two evaluations were performed consecutively on the same day by two researchers in order to determine the interobserver agreement. A third evaluation was carried out by one of the initial researchers after an interval of 7 to 14 days in order to determine the intraobserver agreement. A questionnaire addressing sociodemographic issues was also applied at this stage in order to characterize the sample.

### External validity

The external validity of SHAQ was tested through correlations with other clinical parameters: quality of life, using the short form-36 (SF-36) questionnaire;^14^ upper limb function using the Disabilities of the Arm, Shoulder and Hand (DASH) questionnaire;^15^ and overall function, using the HAQ,^7^ in the second evaluation.

### Statistical analysis

Descriptive statistics (means and standard deviations) were used to analyze the clinical-demographic data on the patients. Pearson's correlation coefficient and the intraclass correlation coefficient (ICC) were calculated in order to evaluate the interobserver and intraobserver agreement. Pearson's correlation coefficient was also used, in order to determine the external validity.

## RESULTS

In the cross-cultural adaptation, two items were not understood by more than 20% of the patients and were changed. In the first item, the patients had difficulty regarding the term "Raynaud's phenomenon" and the following was added to explain the meaning of this term: "fingers that alternate in color between purple, paleness and red, due to the cold". In the second item, "digital ulcers" was replaced by "sores on the fingers". Only these two modified itens were then applied to a new group of 20 patients with systemic scleroderma and were understood by more than 80% of the sample.

*Assessment of measurement properties*

### Characterization of the sample

Table 1 displays the clinical and demographic characteristics of the 20 patients who participated in determining the reliability and validity of SHAQ. All of these 20 patients had a diagnosis of diffuse systemic scleroderma. The mean duration of symptoms was 10.7 years (range: 2 to 36 years) and the mean time that had elapsed since diagnosis was 7.1 years (range: 1 to 19 years).

**Table 1:** Clinical and demographic characteristics of the 20 patients who participated in determining the reliability and validity of the Scleroderma Health Assessment Questionnaire

Clinical-demographic data	
Gender – F/M	18/02
Race (n %)	
White	11 (55)
Nonwhite	9 (45)
Age - mean (SD)	43.7 (11.7)
Marital status (n %)	
Married	10 (50)
Single	5 (25)
Others	5(25)
Education level - n (%)	
Incomplete elementary education	9 (45)
Completed elementary education	4 (20)
Others	6(35)
Duration of symptoms, years - mean (SD)	10.7 (8.9)
Time since diagnosis, years - mean (SD)	7.1 (4.5)

F = female

M = male

SD = standard deviation

### Reliability

In the interobserver evaluation, Pearson's correlation coefficient and the intraclass correlation coefficient were both 0.967 and were considered statistically significant (P < 0.001). The paired Student's t test did not show any statistically significant differences in the findings between the two observers (P = 0.320).

In the intraobserver evaluation, Pearson's correlation coefficient was 0.735 and the intraclass correlation coefficient was 0.687. These results were also considered statistically significant (P < 0.001). The paired Student's t test did not show any statistically significant differences in the findings between the two different evaluations (P = 0.055) (Table 2).

**Table 2:** Interobserver and intraobserver reliability

Reliability		
	Interobserver	Intraobserver
PCC (P value)	0.967 (< 0.001)	0.735 (< 0.001)
ICC (P value)	0.967 (< 0.001)	0.687 (< 0.001)

PCC = Pearson's correlation coefficient

ICC = intraclass correlation coefficient

*External validity*

To determine the external validity, SF-36,^14^ DASH^15^ and HAQ^7^ were administered to 20 patients. SHAQ scores were statistically associated (P < 0.05) with all measurements, except the general health domain of SF-36 and the work-related score (Q2) of the DASH questionnaire (Table 3).

**Table 3:** Pearson's correlation between Scleroderma Health Assessment Questionnaire (SHAQ) and short form-36 (SF-36), Health Assessment Questionnaire (HAQ) and Disabilities of the Arm, Shoulder and Hand (DASH)

Questionnaires	Score - mean (SD)	Correlation with SHAQ	
SHAQ	0.910 (0.681)	Pearson’s correlation	P-value
SF-36			
Physical function	63.8 (28.1)	-0.843	< 0.001
Role - physical problems	47.5 (45.1)	-0.684	0.001
Body pain	68.2 (30.5)	-0.839	< 0.001
General health	59.1 (20.1)	-0.436	0.054
Vitality	52 (50)		
Social function	83.1 (27)	-0.646	0.002
Role - emotional problems	63.3 (45.8)	-0.565	0.009
Mental health	63.8 (29.5)	-0.656	0.002
DASH			
Component 2: work	17.9 (25.9)	0.546	0.128
Component 3: overall score	25.9 (21.5)	0.948	< 0.001
HAQ	0.844 (0.749)	0.96	< 0.001

Values expressed as mean (standard deviation, SD).

## DISCUSSION

Two options are available for obtaining new health assessment measurements: developing a new assessment tool that addresses the issue in question; or translating an existing questionnaire that has demonstrated adequate applicability in other countries. The former is considered to be time-consuming and labor-intensive. Thus, translation of good-quality questionnaires is preferable, since the evaluation should be universal, thus enabling comparison of data from one population with data from other populations throughout the world.^15^

A number of questionnaires in Portuguese have been administered to patients with chronic diseases, in order to assess function,^7^ ^,^ ^15^ ^,^ ^16^ but none of them are specific to the symptoms of systemic scleroderma. Since SHAQ is a valid, reliable questionnaire^17^ ^,^ ^18^ and has been used in a number of countries,^9^ ^,^ ^10^ this questionnaire was chosen for translation into Brazilian Portuguese in the present study.

An internationally accepted method that was specially created to systematize translation of questionnaires was used in this study.^11^ ^,^ ^12^ This is the only way to ensure that the translation presents an equivalent context, in comparison with the original questionnaire. However, translation alone is insufficient for ensuring that a given questionnaire is reliable. It is also necessary to perform cross-cultural adaptation of the text, which may undergo modifications while respecting the initially proposed context and ideas.^11^ In the present study, the entire translation and cross-cultural adaptation process was carried out at our institution' outpatient clinics. Most of the patients in these clinics had lower socioeconomic and schooling levels, thereby representing an important portion of the Brazilian population that receives medical care in this and other reference centers throughout the country.

Given the low schooling level of the patients in the present study, SHAQ was applied in interview form, which was done in order to increase the number of people on whom the questionnaire could be used. This method has also been used in previous studies.^7^ ^,^ ^14^ ^,^ ^15^ In the study by Steen et al.,^9^ who created SHAQ, and Georges et al*.*,^10^ who validated the French version, the questionnaires were self-administered.

HAQ has been used to assess overall physical function and has items addressing different activities of daily living, such as eating, having a bath and walking. Poole and Steen^19^ administered HAQ to patients with systemic scleroderma and concluded that this measurement was inadequate for assessing all the symptoms and manifestations of the disease. Thus, Steen and Medsger added five visual analogue scales (VASs) to HAQ, thereby creating SHAQ,^9^ which was validated in France in 2003.^10^ The addition of these five VASs addressing the symptoms of systemic scleroderma makes SHAQ a specific questionnaire that assesses function in patients with this disease.^18^ The VAS relating to Raynaud's phenomenon was not validated in the original questionnaire, but was administered in the study by Georges et al*.* ^10^ in order to validate the French version of SHAQ.

Both HAQ and SHAQ have been applied together in order to compare data and determine the validity of the five VASs that were added to the former.^18^ This author reported that both questionnaires were sensitive for assessing difficulties in performing activities of daily living, among patients with systemic scleroderma, and that SHAQ enhances HAQ, since it assesses the impact of the symptoms of the disease on daily activities.

Following translation, the Brazilian version of SHAQ was found to contain technical terms that were not widely known by the general population and were therefore difficult to understand. Thus, the terms *Raynaud's phenomenon* and *digital ulcers *were changed without altering the meaning of the items. In contrast, no changes had to be made to the French version of the SHAQ.^10^

In characterizing the sample, the mean age of the 20 participants was 43.7 years; 90% were women and 10% were men. In the study by Georges et al.,^10^ the mean age was 50 years; 76% of the participants were women and 24% were men. The female gender predominates because this disease affects women more.^3^

A high level of agreement was found in assessing interobserver reliability, whereas a moderate level of agreement was found in assessing intraobserver reliability. This finding may be attributed to the fact that intraobserver reliability was determined with a 14-day interval between evaluations. Some symptoms of systemic scleroderma are dependent on the weather, such as Raynaud's phenomenon and lung problems. Changes in the weather within a two-week timeframe are enough to change the symptoms of the disease, which could change the VAS scores, thereby affecting the reproducibility of SHAQ. However, in the study by Georges et al.,^10^ the interval between the assessments for determining intraobserver reliability ranged from one week to one month, and the authors reported a test-retest coefficient of 0.98, even with the large time interval between evaluations. Despite this difference, the Brazilian version of SHAQ proved to be reproducible.

Quality of life was assessed using the SF-36 questionnaire. The scores on the functional capacity, role-physical, pain, role-emotional, vitality, social aspects and mental health subscales demonstrated strong correlations with the SHAQ score. The only non-significant correlation was with the general health state subscale. Correlations between these two measurements were expected, since physical impairment is often associated with reductions in both physical and mental health in patients with systemic scleroderma.^20^ Moreover, a systematic review has reported that symptoms of depression are found in 36 to 65% of such patients and are strongly associated with joint pain as well as the duration and severity of the disease.^21^ In the study by Georges et al.,^10^ all the SF-36 subscales were statistically correlated with the SHAQ score. With chronic disease, inactivity stemming from pain leads to a greater risk of accelerated loss of muscle mass, with consequent reduction in muscle strength and diminished level of independence, thereby contributing towards a reduction in quality of life.^22^

HAQ was also statistically correlated with SHAQ, and the P-value demonstrated that the scores were directly proportional. This finding was expected, since SHAQ is the same as HAQ with the addition of the five VASs. The mean score for the questionnaire was 0.844. In a study by Singh et al.,^23^ which assessed productivity among patients with systemic scleroderma, HAQ for patients with the diffuse form of the disease was 1.2 (SD = 0.7). It needs to be borne in mind that higher scores are associated with a lower degree of productivity.^23^ Georges et al.^10^ found a very strong correlation between the two questionnaires, as well as strong correlations between these questionnaires and the severity of the disease.

The DASH questionnaire is commonly used to assess upper-limb conditions and was used in the present study because thickening of hand skin is one of the most common symptoms of systemic scleroderma and the reason for loss of hand function. This questionnaire proved to be correlated with SHAQ. In the present study, the score for component 1 was not calculated, since this component was only relevant to two patients, which rendered statistical description impossible.

Higher SHAQ scores were found in the present study than in the study by Georges et al.,^10^ who reported a mean score of 0.02. In the present study, all the patients had diffuse systemic scleroderma, which affects internal organs and commonly presents symptoms of greater severity than is seen in cases of limited scleroderma, thereby increasing these patients' dysfunction and consequently leading to higher scores in questionnaires that assess this variable. In the study by Georges et al.,^10^ 77% of the patients had diffuse systemic scleroderma and the remaining patients were classified as presenting the limited form of the disease, which may explain the difference in scores between the two studies.

Disease duration was similar in the two studies. In the present study, the mean duration of symptoms was 10.7 years, which may explain the low scores found in evaluating upper limb function using DASH, since patients tend to develop adaptations to maintain their activities of daily living. This may also explain the low score on the "role-physical" subscale of the SF-36 questionnaire. The majority of the patients reported typical symptoms of systemic scleroderma throughout the present study, such as Raynaud's phenomenon, respiratory and digestive problems, digital ulcers and thickening of the skin, which affect the SHAQ score. In the study by Georges et al.,^10^ 68% of the patients had Raynaud's phenomenon in the final week; approximately 50% had respiratory, digestive and heart problems and only 25% experienced contractures in the hands. These findings may explain the differences in SHAQ scores between the two studies.

Although the original questionnaire was tested for both forms of progressive systemic scleroderma, the Brazilian version was produced only using patients with diffuse systemic scleroderma. The Brazilian version probably maintains the original measured performance, but it needs to be tested on cases of limited scleroderma.

Previous studies have used other questionnaires that assess function, but these questionnaires are not specific to systemic scleroderma. Thus, validation of SHAQ enables a more specific evaluation of function, directed towards patients with this disease, thereby making this questionnaire an additional assessment tool of considerable value. The successful translation and cross-cultural adaptation of SHAQ will allow future studies to assess function in patients with systemic scleroderma and test the responsiveness of the questionnaire. Moreover, the use of this measurement in different populations allows comparisons of the findings across cultures.

## CONCLUSION

In conclusion, the Brazilian version of the Scleroderma Health Assessment Questionnaire proved to be a valid, reliable measurement tool for assessing function in patients with diffuse systemic scleroderma.
